# Stochastic coastal flood risk modelling for the east coast of Africa

**DOI:** 10.1038/s44304-024-00010-1

**Published:** 2024-06-03

**Authors:** Irene Benito, Jeroen C. J. H. Aerts, Dirk Eilander, Philip J. Ward, Sanne Muis

**Affiliations:** 1 Institute for Environmental Studies (IVM), VU University Amsterdam, Amsterdam, The Netherlands; 2 Deltares, Delft, The Netherlands

**Keywords:** Climate sciences, Natural hazards

## Abstract

Coastal flooding resulting from tropical cyclones can have large repercussions in many low-lying regions around the world. Accurate flood risk assessments are crucial for designing measures to reduce the societal impacts of coastal flooding. At continental to global scales, however, traditional flood risk assessments mostly use methods that do not capture the spatiotemporal dynamics of coastal flood risk patterns. In this study, we address these deficiencies by applying a novel modelling framework that dynamically simulates stochastic coastal flood risk for the east coast of Africa. Using 10,000 years of synthetic tropical cyclones and a cascade of hydrodynamic models to simulate storm tides and flooding, we calculate the damage of each individual tropical cyclone event and empirically derive the risk curve for each country. Results show that the largest aggregated annual losses in the region come from multiple events rather than from a single low-probability event. Results also reveal that events with the highest return periods in terms of storm surge residual levels and flood extents are not necessarily the most damaging events. Here, the 1 in 10,000-year damage event is associated with a 1 in 45-year event in terms of flood extent, showing that addressing exposure and vulnerability is essential in determining risk. Our modelling framework enables a high-resolution continental-scale risk analysis that takes the spatial dependencies of flood events into account.

## Introduction

Extreme coastal flood events can have devastating impacts in densely populated and low-lying coastal areas, affecting societies, economies, and the environment^1^. Flood risk assessments can play a key role in reducing the potential impacts of these events. At local to regional scales, this information is used for the design of flood protection or early warning systems^1–4^. At the continental to global scales, coastal flood risk assessments can be used for pricing insurance and (re-)insurance policies, identifying risk-prone areas, and understanding flood hazard and risk in data-scarce regions^5–8^.

Flood risk is characterised by three components: hazard, often quantified as the flood depth at different return periods derived from parametric or empirical extreme value distributions; exposure, defined in terms of economic assets and population; and vulnerability, representing the susceptibility to the impacts. Flood risk is commonly calculated by integrating the flood impacts over their probability of occurrence to derive annual expected damage^9^. To compute risk, coastal flood risk assessments use a chain of different models. First, hydrodynamic models are used to simulate the extreme water levels along the coast, that occur as a result of the combination of tides, storm surges, and waves^10–12^. Second, flood models, driven by extreme water levels, are used to simulate flood depth and extent in coastal regions^13–15^. Last, impact models are used to calculate the economic damages and risk^13–15^. At continental to global scales, the traditional approach of coastal flood risk assessments is to calculate the return periods of coastal water levels by using extreme value statistics^10,16,17^. Extreme water levels associated with a specific return period, for example, the 1 in 100-year event, are then used to obtain flood hazard maps associated with that return period. This traditional approach assumes full dependence on extreme water levels along entire coastlines^18,19^ and neglects the physics and duration of flood events^13,20^. At large scales, these simplifying assumptions can result in under/over-estimations of the flood hazard that propagate into the risk assessments and can, therefore, lead to an unrealistic representation of the risk^13,18–23^.

Stochastic risk modelling can be used to address the full spatial dependence assumption of traditional approaches by simulating a large number of stochastic events with realistic spatiotemporal patterns^18,24^. Risk is then quantified following a Monte Carlo rather than a deterministic method. Applying a stochastic approach, the dynamic modelling does not stop at the coastline with the simulation of water levels. Instead, the propagation of the water further inland is dynamically modelled to obtain flood hazard maps. Subsequently, flood damages are calculated for each event. The extreme value statistics are then derived empirically based on the damages instead of fitting a distribution to the water levels, as is done in the traditional approach. The stochastic approach requires the simulation of thousands of possible flood events based on certain climate conditions. Historical climate data typically cover a short time period of up to ~50 years^25^, which does not provide a sufficient number of extreme events for stochastic risk modelling. To overcome this drawback, recent developments have focused on creating synthetic event sets derived from either large climate ensemble datasets^26,27^ or from the use of statistical models^28–33^.

Until now, the application of stochastic approaches for coastal flood risk assessments at large scales has been limited by its large computational expenses. In this study, we address that limitation by introducing an innovative and efficient modelling framework. This framework allows us to compute high-resolution flood modelling at continental scale using a large set of stochastic events at reasonable computational costs. We accomplish this by using 10,000 years of synthetic tropical cyclone (TC) events (122,544 TC events) from the Synthetic Tropical cyclOne geneRation Model (STORM)^28^ combined with the Global Tide and Surge Model (GTSM)^10^ to simulate coastal water level timeseries. Next, we model the flooding caused by each event by linking various hydrodynamic SFINCS (Super-Fast INundation of CoastS)^34^ models. Subsequently, we calculate the damage of each event by combining its flood depth hazard map with exposure datasets, including population count maps and build-up area maps, and depth-damage curves. Last, we derive the return periods associated with the damages and annual losses based on their empirical distribution, which is possible due to the large sample size of damages as a result of 10,000 years of events.

To demonstrate the potential of the modelling framework, we focus on coastal flood risk driven by TC-induced storm surges, using the east coast of Africa as a case study. This study area covers several countries which are prone to TCs coming from the Indian Ocean, and that belong to the South Indian TC basin: Mozambique, Tanzania, South Africa, Madagascar, Comoros, Reunion (FR Territory), Glorioso Islands (FR Territory), Mayotte (FR Territory), Mauritius and Seychelles. On average, these countries experience 1 to 4 cyclones per year^35^. According to ref. ^16^, the combined potential hazard of TCs and extratropical cyclones of a 1 in 1000-year water level event is approximately 5 metres in Mozambique and 3.5 metres in Madagascar. This region has been affected by several devastating TC events in recent years, including TC Idai in 2019. This event caused large storm surges in the city of Beira in Mozambique, and resulted in more than one hundred fatalities and economic losses of up to US $3.3 billion^36^. The large size of the TC basin makes it a good case study, as it allows for capturing the spatial variability of TC events, necessary to examine the added value of stochastic flood risk modelling approaches.

## Results

### Frequency analysis

**Fig. 1:**
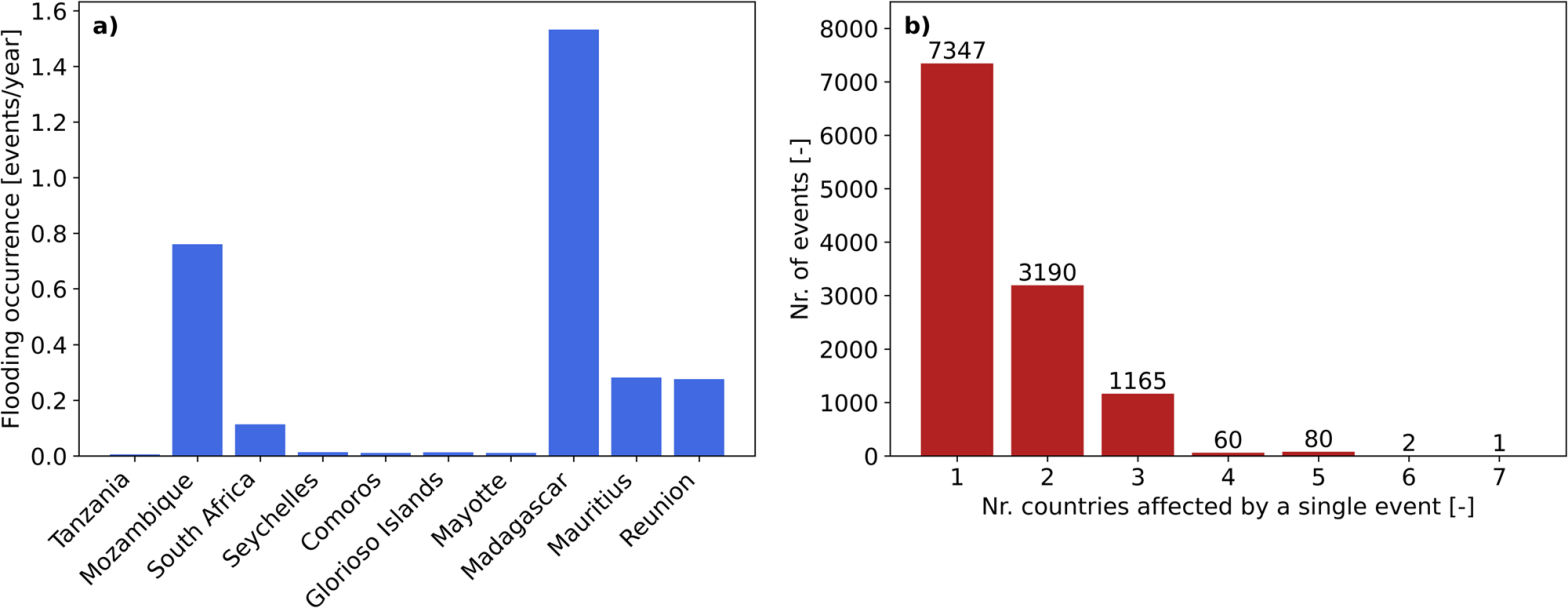
Frequency analysis. **a** number of flood events per year per country; and **b** frequency diagram with the number of countries affected by single events.

The simulated annual flood frequency of coastal flooding from TC events on the east coast of Africa is shown in Fig. 1a. Madagascar and Mozambique are the two countries with the highest flood frequency. On average, Madagascar and Mozambique experience, respectively, 1.5 flood events and 0.75 flood events per year. Other countries, including Mauritius and Reunion, have flood frequencies in the order of 0.3 events per year. Figure 1b shows the number of countries affected by a single event. Out of the 11,845 events that cause damage, the majority of the events (i.e., 62%) have an impact in one country, while 0.02% of the events affect >5 countries. Events affecting Mozambique, typically also have an influence in Madagascar. Out of the 2475 events in Mozambique, 44% also cause damages in Madagascar.

### Risk analysis at country scale

**Fig. 2:**
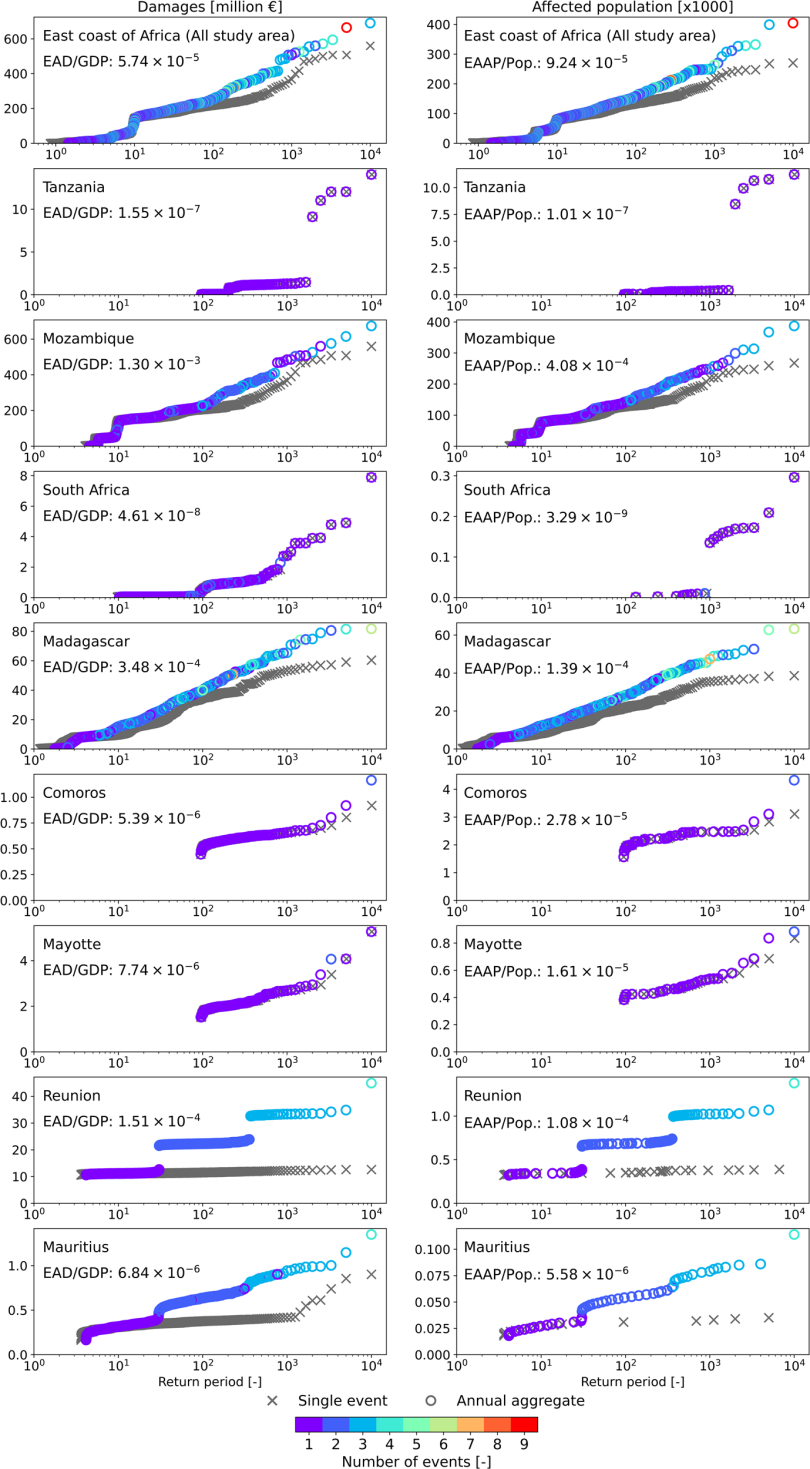
Loss-exceedance curves for all the study area (top row) and for each separate country. Left: loss-exceedance curves for economic damages, and the expected annual damages (EAD) over gross domestic product (GDP). Right: Loss-exceedance curves for affected population, and the expected annual affected population (EAAP) over total population (Pop.). The grey crosses indicate the return periods of each event that caused damage. The circles indicate the return periods of the aggregated annual damages, being the colour of each circle associated with the number of events that caused damages on that specific year.

Next, we analyse the probability distributions of damages and affected population. Figure 2 shows the single event and aggregated annual loss-exceedance curves for the whole study area and for each specific country within this region. The 1 in 100-year damage level for the east coast of Africa is 254 million euros and 135k of people affected, while the 1 in 1000-year damage level results in 367 million euros and 220k people affected. The results show that along the east coast of Africa, the years with the highest damages are not associated with a single low-probability event but are the result of multiple events in a single year, which may not necessarily be the most damaging events. The year with the highest aggregated annual economic damages along the east coast of Africa occurs in a year when three events hit the region, while the year with the most affected population is the result of nine aggregated events. The expected annual damage (EAD) for the entire study region is 30 million euros, and the expected annual affected population (EAAP) is 17.7k people.

Figure 2 provides the EAD with respect to the gross domestic product (GDP) of each country, and the percentage of EAAP with respect to the total population of each country. It shows large differences in flood risk among the different countries. Mozambique is the most impacted country in the study area. The EAD in Mozambique is 0.13% of its GDP, and 0.04% of its population is annually exposed to floods. Madagascar and Reunion also show significant EADs with respect to their GDP, being 0.03% and 0.02% of their GDPs, respectively. 0.01% of the population of both countries is also annually exposed to floods. For each country, the loss-exceedance curves follow similar trends for damages and affected populations. However, the shape of the curves differs significantly among countries. While for Tanzania and South Africa, the highest aggregated annual damages are caused by single events, for Reunion and Mauritius, the highest aggregated annual damages are caused by multiple events, namely four events in a single year. The aggregated annual loss-exceedance curves of Reunion and Mauritius show sharp discontinuities, which are caused by the shift in the number of events occurring in each aggregated year.

### Worst case events

Out of the 11,845 events that caused impacts along the east coast of Africa, the five most damaging events hit Mozambique, of which one also hit Madagascar. Of these five events, four have category 2 on the Saffir-Simpson scale at landfall, and all five make landfall in the surroundings of Beira. All events generate water levels higher than 4.5 m, causing flood extents of >1000 km^2^. The consequences of each of those flood events are losses of >485 million euros and >240k people affected.

**Fig. 3:**
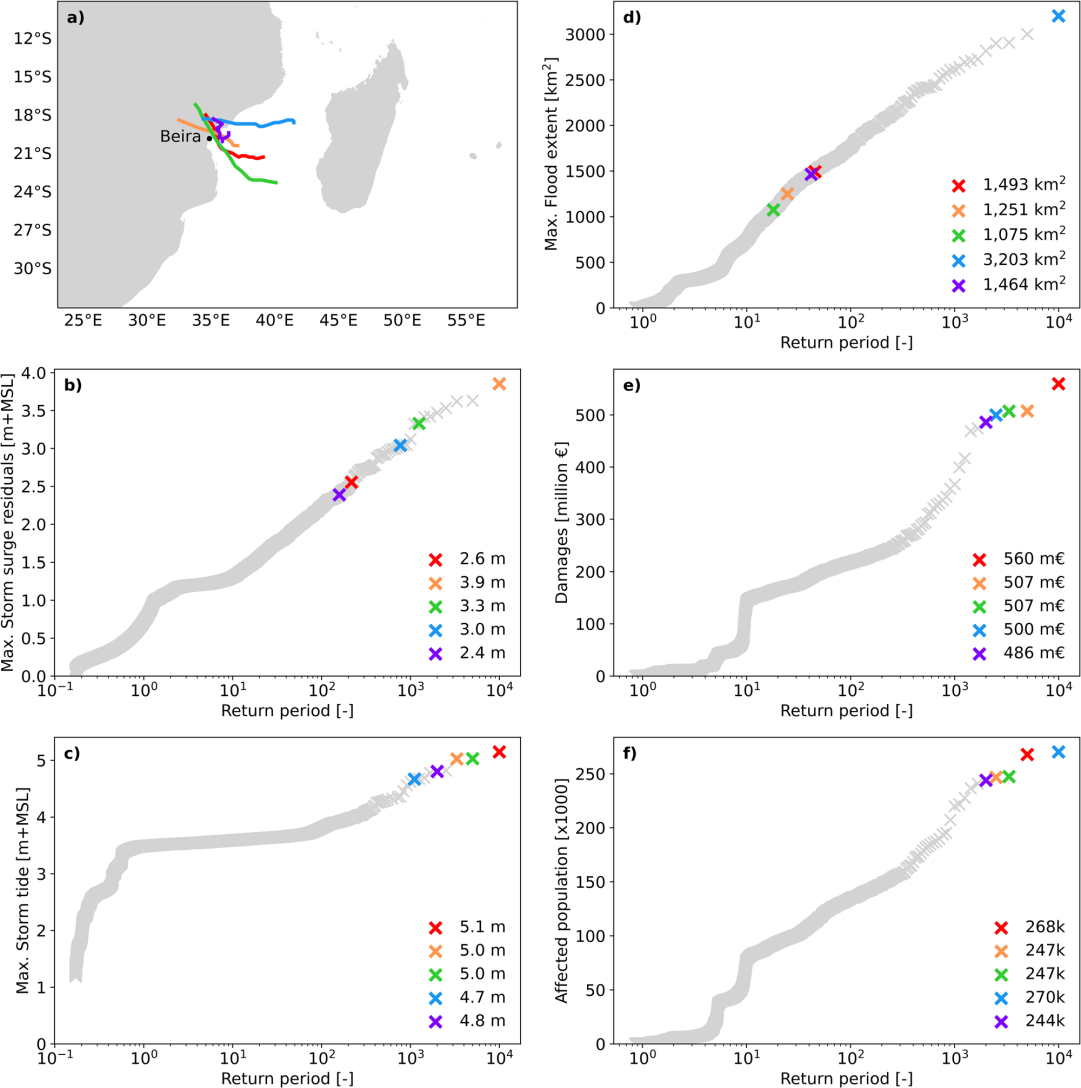
Return period curves for different metrics. **a** TC tracks of the 5 most damaging events. Each colour track represents an event. Plots **b**–**f** provide the cumulative distribution functions of storm surge residuals (**b**), storm tides (**c**), flood extents (**d**), damages (**e**), and affected population (**f**). Grey crosses indicate all the events within the study area, while the colored crosses are associated with each of the five most damaging events from **a**.

The return periods of the events in the 10,000 years dataset are shown in Fig. 3. The return period in the different panels (b-f) is shown in terms of the storm surge residuals (difference between the storm tide and the predicted astronomical tide), storm tide, flood extent, damages, and affected population, respectively. The worst five events in terms of economic damages are displayed in colours, while the other events are shown in grey. It can be observed that the return periods associated with the storm surge residuals (panel b) do not match the return periods associated with the storm tides (panel c). Some events with lower storm surge residual return periods coincide with spring tide, resulting in higher water levels. On the other hand, higher return period storm surge residuals occur in combination with lower tidal levels. As a result, the most damaging events have similar storm tides for storm surge residuals of different probabilities. The 1 in 10,000-year event in terms of water levels results in the most damaging event (panel e, red cross), even though it is associated with a 1 in 217-year event in terms of storm surge residuals (panel c) and a 1 in 45-year event in terms of flood extents (panel d). This shows that the exposure and vulnerability of the locations where the flood occurs are essential in determining the damage of an event. In urban areas, the location of economic assets and population generally overlap. Because of this, the most damaging events are also the ones that affect the highest number of people.

## Discussion

This study introduces a novel and optimised modelling framework that uses hydrodynamic models to simulate water levels and high-resolution flooding on an event-by-event basis at a continental scale, and performs extreme value statistics of their damages. This modelling framework allowed us to produce the first-to-date stochastic coastal flood risk assessment for the east coast of Africa. The results of this assessment show that multiple events contribute to the aggregated annual losses in our study area, and that moderate hazard events can become extreme impact events depending on their landfall location and timing with tides. In this section, we discuss several limitations which may influence our results.

We focus on coastal flooding induced by storm surges, though other drivers like waves, rainfall, or river discharge can cause flooding during TC events. Our modelling framework uses GTSM to simulate water levels resulting from TCs. GTSM is a global depth-averaged hydrodynamic model that has been shown to perform well when forced with TC tracks^37,38^. However, this model lacks resolution in regions with complex topographies^39^. While we start our GTSM simulations at 100 random times, reflecting different tidal initial conditions, those might not be enough to cover the whole spectrum of potential tide-surge combinations. This could potentially be improved by using a larger sample size of initial conditions.

To apply an empirical extreme value analysis in a stochastic approach, it is necessary to first define what an event is. In this study we define events based on TC tracks that generate impact either in terms of economic damage or affected population. Therefore, TC events that cause no impact are excluded from our analysis. When defining events differently, the probabilities would change. The aggregation of risk at different levels of administrative units would also change the risk analysis. Furthermore, in small countries such as the Seychelles and Glorioso Islands few TCs make landfall. Those TCs that make landfall, however, cause flooding in areas where there is no exposure, and hence, the resulting risk for those two regions is zero. However, such results should be interpreted with caution due to the following modelling limitations. The exclusion of discharge and mean sea level variations in our study can have an effect on the water level estimates. Our results are based on the most up-to-date global datasets. However, these datasets might be too coarse to resolve local processes. The bathymetry data is a source of uncertainty that can have a large effect on the simulated water levels, and inaccuracies in the global DEM can have direct implications for the simulated flooding. A large limitation of our study is the exclusion of flood protection levels due to a lack of data. Literature suggests flood protection levels of 2–25 years on the east coast of Africa^40^, but it also highlights the importance of Nature-based Solutions for flood protection, such as mangroves^41,42^. ref. ^43^ reported that at present day, the east coast of Africa has a reduction of EAD US $7.8 million thanks to mangroves.

A major challenge of modelling synthetic events is the validation of the results. Firstly, because those events did not actually occur. Hence, it is not possible to assess the accuracy of our modelled events by comparing them one to one with historical events. Secondly, when looking at resemblance with historical events, the losses reported for an event usually include direct and indirect damages as a result of multiple flood drivers, such as pluvial, fluvial, or coastal flooding, but also wind damages. Therefore, it is not possible to isolate the direct impact and risk of coastal flooding alone. We performed our simulations with validated models that showed good modelling performance, and our results are in good agreement with other studies that modelled total water levels and flooding in the region^16,44^. However, it is known that STORM underestimates wind speeds in the South Indian basin^45^. This could lead to an underestimation of the storm surges we modelled, and hence, the risk estimated for the region.

A stochastic approach like we propose in this paper adds value over traditional coastal flood risk approaches, because it takes into account the spatial and temporal variability of events^18,24^. This allows for analyses that are not possible on traditional risk assessments. For example, stochastic approaches allow deriving frequencies and spatial magnitudes of events. They also allow for deriving loss-exceedance curves associated with events. These, combined with loss-exceedance curves of aggregated annual damages, can help to better understand risk and obtain information about the source of the aggregated annual damages. Our results show that aggregated annual damages can be caused by either a single low-probability high-impact event or by multiple events. Depending on the source of those annual damages, governments, and (re-)insurance companies will determine their preventive measures and risk portfolios differently. These exceedance curves also provide information about the extreme events located at the tail of the probability distributions. The (re-)insurance sector is interested in the impacts of specific high-impact events. This information can also offer valuable insights to enhance emergency preparedness, risk mitigation strategies and risk financing^46^. Contrary to traditional risk approaches that associate extreme hazards with extreme impacts, we show that the highest storm surge residual levels and flood extents do not necessarily result in the highest damages. For example, here, the most damaging event is a 1 in 217-year event in terms of storm surge residuals and a 1 in 45-year event in terms of flood extent. These results are in line with ref. ^47^ who, in a different context, showed that extreme impacts can result from moderate meteorological conditions.

Future research directions to improve our approach include the implementation of other flood drivers into the assessment other than tides and storm surges. Ref. ^44^, for example, showed the significance of compound flooding in the region of Beira, Mozambique, with rainfall and discharge being important drivers of the overall flooding. Also, wind waves are known to contribute to coastal flooding^48,49^. In the coming years, the frequency of coastal flooding will increase due to the rise in global temperatures, which will cause mean sea level rise and changes in the occurrence of TCs^50^. This, combined with the projected increase in coastal population^51^, will inevitably lead to a change in the risk profile of coastal communities. ref. ^52^ derived a dataset of synthetic TC tracks under climate change using the STORM algorithm. Updating the stochastic approach presented here with those synthetic tracks and including sea level projections for the same climate scenarios would allow assessing the changes in coastal flood risk on the east coast of Africa in response to climate change. Global scale flood risk assessments still use return periods associated with water levels and planar flood modelling approaches, neglecting the spatiotemporal variability of coastal flooding events. Another future research direction would be the upscaling of the current approach to global coverage. Since we used global datasets in this study, such upscaling seems within reach, with the high computational costs being the main constraint.

This study presents a framework that dynamically simulates water levels and flooding, allowing the modelling of stochastic high-resolution coastal flood risk at continental scales. The outcomes of this research can be applied in the development of risk reduction measures, aid funds and insurance portfolios. Understanding better the risks of coastal flooding can reduce its potential impacts thanks to improved decision making and emergency strategies. Furthermore, the modelling framework presented in this study could be highly useful in the development of stochastic risk assessments for other regions.

## Methods

### General approach

**Fig. 4:**
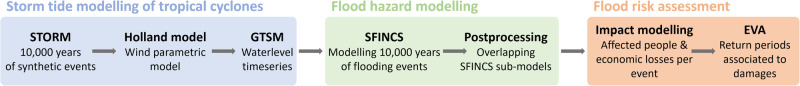
Modelling workflow. Stochastic approach used to perform the coastal flood risk assessment.

In this study, we present a novel approach for stochastic risk assessment of storm surge-induced flooding due to TCs using a modelling framework. Figure 4 shows the workflow for this approach. This new stochastic flood risk approach consists of three main steps: (1) storm tide modelling of TCs; (2) flood hazard modelling; and (3) flood risk assessment.

Traditional flood risk approaches use water levels associated with return periods to simulate flooding with a planar approach. Subsequently, an impact model is used to calculate the damages for specific return periods. Stochastic approaches, on the other hand, calculate the damages of each event individually to finally derive the return periods associated with damages.

The computational expenses of this study are ~230,400 core hours, with 70% of allocated to the storm tide modelling and 30% to the flood hazard and risk modelling.

### Storm tide modelling of tropical cyclones

The modelling of storm tides as a combination of storm surges and tides consists of three main steps. As input, we use the STORM to simulate the TC-induced storm tides of 10,000 years of synthetic TC events. STORM is a global dataset representing 10,000 years of TC events under present climate conditions, based on historical TC tracks^25^ and following a fully statistical approach^28^. The TC variables provided by STORM include maximum windspeed, mean sea level pressure, longitude, latitude, and radius to maximum winds at a 3-hourly resolution.

As a first step, we filter the STORM dataset to reduce the runtime of the simulations. For this purpose, we select only TC events in the South Indian TC basin that could generate a storm surge relevant to the coastal areas of our study. Therefore, only TC tracks that come within 750 km distance from land are selected. For each of those TC tracks, only the part of the track within 1000 km from land is used^16^. This filtering approach allows for reducing the event sample from 122,544 to 58,304 events for the 10,000 years of the STORM dataset.

The second step converts the TC tracks from STORM into wind and pressure fields. For this, we use the Holland parametric model^53^. TC fields are defined following the approach of ref. ^16^, with a 750 km radius, 375 arcs, and 36 radial bins. The surface background wind is derived from ref. ^54^, where the reduction factor and angle of surface background wind are 0.55 and 20°, respectively. A surface wind reduction factor (SWRF) of 0.85 is used to translate the gradient wind to surface level^55^.

**Fig. 5:**
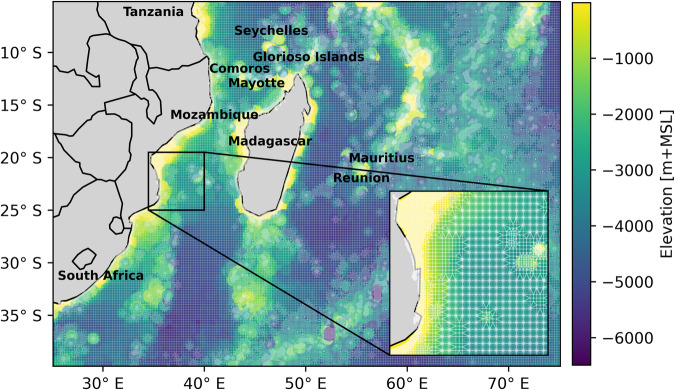
Model domain of the GTSM. Unstructured mesh grid with a resolution of 25 km in the deep ocean and 2.5 km along the coast.

The third step simulates the TC-induced storm tides using a regional cut-out of the Global Tide and Surge Model Version 4.1 (GTSMv4.1). GTSMv4.1 is a calibrated depth-averaged hydrodynamic model of global coverage based on Delft3D Flexible Mesh^10,56,57^. GTSM has demonstrated good performance in simulating accurate water levels when forced with track data^16^, and has been successfully applied for a case study of Mozambique by ref. ^44^. The grid resolution of the model varies from 25 km in the deep ocean to 2.5 km along the coast (1.25 km in Europe). To optimise the runtime, we generate a regional model based on the global model covering the domain from a latitude of 40°S to 5°S, and longitude from 25°E to 75°E (see Fig. 5). To improve the water level simulations, we upgrade the bathymetry from the default General Bathymetric Chart of the Ocean (GEBCO) 2014 with 30 arc-second interval grid to the General GEBCO 2020, with a 15 arc-second interval grid^58,59^. For each TC, the model is forced with the wind and pressure fields from the Holland model. The wind stress is simulated using the formulation of ref. ^60^, setting the maximum drag coefficient to 0.0025. To account for the tidal forcing, the tidal constituents from the Finite Element Solution global ocean tidal atlas are used as boundary conditions^61^. Finally, to obtain better coastal boundary conditions for the flood model, the output resolution of GTSM along the coast is enhanced up to ~2 km (compared to ~5 km in the global model). To simulate the associated water levels of TC events, each TC event is modelled in GTSM separately with a random tide associated with it. The nodal cycle, as reported by ref. ^62^, and seasonal variabilities in sea surface height, as reported by ref. ^63^, are both <30 cm in our study area. Given the small contribution of these processes to the total water levels and their associated increase in computational expenses, they are not considered in the simulations. In addition, monthly fluctuations in tidal levels are determined, which show that the majority of observations exhibit an average standard deviation of less than 15 cm for the maximum and minimum tidal levels (Supplementary Fig. 1). Therefore, a random month of a random year is selected within the TC season in the selected basin. Each model run is then initiated at a randomly selected hour from a pre-defined set of 100 hours within that month, a measure taken to minimise storage requirements. To minimise the computational time required to spin up each simulation (which has a duration of about 2 weeks) we use a 1.5-month tide-only simulation as initial condition for each simulation.

### Flood hazard modelling

In order to simulate the coastal flooding caused by storm tides, we use the Super-Fast INundation of CoastS (SFINCS) model forced with the storm tide timeseries output from GTSM (“Storm tide modelling of tropical cyclones” section within “Methods”). SFINCS uses simplified equations of mass and momentum, yielding a more computationally efficient dynamic flooding approach than full shallow water equation models^34^. These equations are based on the LISFLOOD-FP model^64^, which uses the Local Inertia Equations to solve the momentum equations for overland flows. SFINCS has been developed for compound flooding, being able to combine fluvial, pluvial, tidal, wind and wave-driven forcings. Its modelling output results in similar results to those from full shallow water equation models, while reducing computational cost by a factor of 100^34^.

As input dataset for elevation to SFINCS, we use FABDEM^65^, a global digital elevation model at 30 m resolution, which has shown great performance for hydrological and flood modelling purposes. To account for the roughness, a spatially varying grid based on land-cover data from the Copernicus Global Land Service is used^66^. For each TC event, SFINCS is forced with the water level timeseries derived with GTSM. The Mean Dynamic Topography (DTU10MDT^67^) is used to convert the vertical reference of the water level dataset from mean sea level to EGM2008 geoid, matching the vertical reference of the elevation datasets. The open-source Python package HydroMT^68^ is used to automate the SFINCS model building, updating the model boundary conditions with GTSM output, and post-processing.

**Fig. 6:**
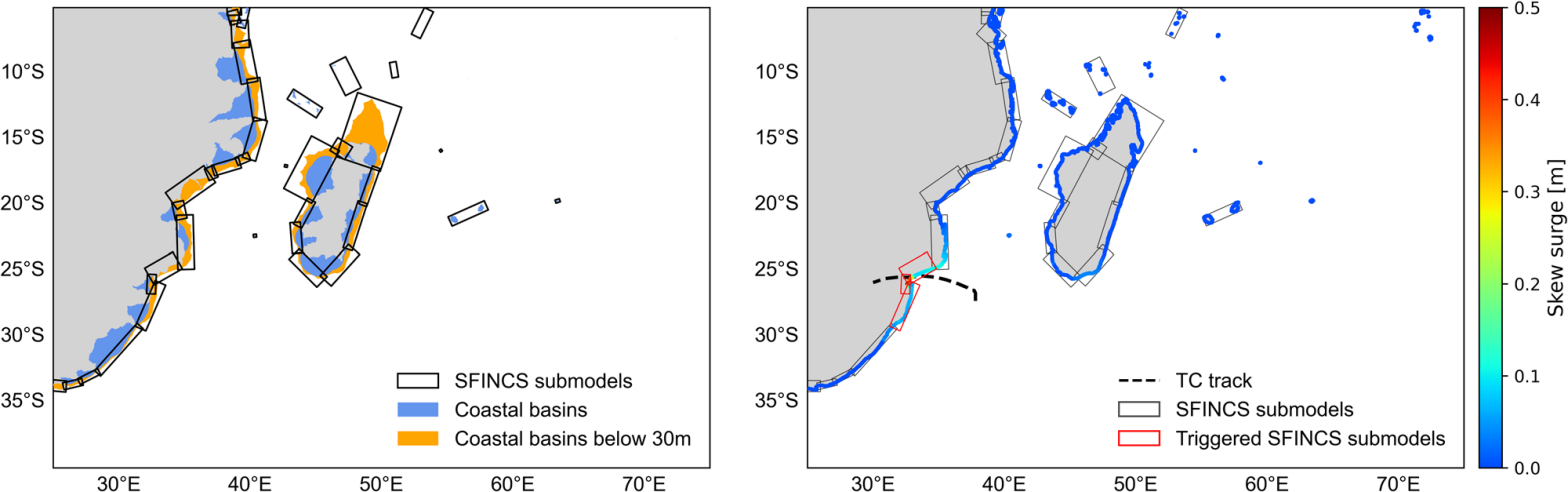
SFINCS model setup. SFINCS models (left) and triggered SFINCS models based on the skew surge for a specific event (right).

To distribute the large computational cost of executing a hydrodynamic flood model for the whole domain of the study and for 58,304 events, the domain is divided into 34 models (see Fig. 6). The SFINCS model domains are automatically generated, taking into account different factors: (1) only regions that are located between 0 and 30 m elevation are included in the models, as higher-elevation regions are not prone to coastal flooding; (2) coastal basins boundaries from HydroBASINS^69^ are used to define the borders of the models; (3) only models with exposed assets and/or population are selected (see “Flood risk assessment” section within “Methods”); (4) the grids of the models are defined within rotated rectangles, in order to reduce the model size and hence minimise the computational times of the flood models; and (5) overlap between the models is defined, in order to allow for better simulation at the borders of the models.

To further speed up the simulations while maintaining the high resolution of the models, the subgrid schematisation recently implemented in SFINCS by ref. ^70^ is applied in each model. A SFINCS model is triggered once it detects within its domain a GTSM output 10 cm above the maximum tidal level of the selected month (skew surge; see Fig. 6).

The maximum flood hazard maps are postprocessed for those events that trigger multiple SFINCS models. Contiguous SFINCS models have an overlapping area. To avoid the double-counting flooded grid cells, the flood depth of one model within the overlapping area is chosen, leaving out a margin to avoid potential simulation issues that may occur at the model edges. The resulting flood maps are used for the flood risk assessment. For the flood hazard maps, all grid cells that exceed 5 cm flood depth are considered flooded.

### Flood risk assessment

Flood risk is calculated using the maximum flood depth of each SFINCS event simulation combined with exposure data and depth-damage curves. To obtain the number of affected people, we use as exposure layer the Global Human Settlement Layer (GHSL) population count maps of the year 2020 at 100 m resolution^71^. We resample the gridded population dataset to the resolution of the 30 m flood hazard maps, using bilinear interpolation of population density. People are considered to be affected when the flood depth on a specific grid cell exceeds 15 cm. To calculate direct economic losses, we use the 10 m resolution built-up maps of 2018 from GHSL associated with residential and commercial uses as exposure layer. Those built-up maps are resampled to the resolution of the 30 m flood hazard maps through the summing method and are combined with the land-use-specific depth-damage functions from ref. ^72^ to calculate direct economic losses. This results in gridded maps of affected population and economic losses.

The damage simulations allow for a further selection of the events. From the 58,304 tracks that have relevant storm tides based on the approach described in the “Storm tide modelling of tropical cyclones” section, 12,708 events cause storm tides larger than the maximum water level of the tidal cycle. However, for the risk analysis, we focus only on those events that cause damages in the study area, resulting in a sample of 11,845 events.

### Analysis of the stochastic approach

We assess the information that stochastic approaches provide. First, we look at the event frequencies in the study region. Next, we use the large sample size with 10,000 years of data to perform an empirical extreme value analysis and calculate the return periods in two manners: return periods associated with aggregated annual damages and affected population, and return periods associated with each event’s damage and affected population in the sample. Risk at country and study area levels is calculated by aggregating the damages in each region. By integrating the total annual losses and affected people, the EAD and EAAP are derived^19,40^. Finally, we focus on the five flooding events in the study area that cause the most economic losses and analyse some of their characteristics.

## Supplementary information

Supplementary Information

## Data Availability

The datasets compiled and/or analysed during the current study are available on Zenodo^73^.

## Code availability

The underlying code for this study is available on Github: https://github.com/Ireneben73/stochastic_risk_east_africa.
