# Supplementary material for: Stochastic coastal flood risk modelling for the east coast of Africa
Source: NPJ Nat Hazards. 2024 Jun 3;1(1):10. doi: 10.1038/s44304-024-00010-1 (PMC12885944; doi:10.1038/s44304-024-00010-1)
Supplement: Supplementary file 1 — Supplementary Information [file 44304_2024_10_MOESM1_ESM.pdf]

Supplementary Information

Stochastic coastal flood risk modelling for the east coast of Africa

Irene Benito[1], Jeroen C.J.H. Aerts[1,2], Dirk Eilander[1,2], Philip J. Ward[1,2], and Sanne Muis[1,2]

1. Institute for Environmental Studies (IVM), VU University Amsterdam, The Netherlands
2. Deltares, Delft, The Netherlands.

Correspondence to: Irene Benito (i.benito.lazaro@vu.nl)

This file includes:

Supplementary Figure 1

Supplementary Figures

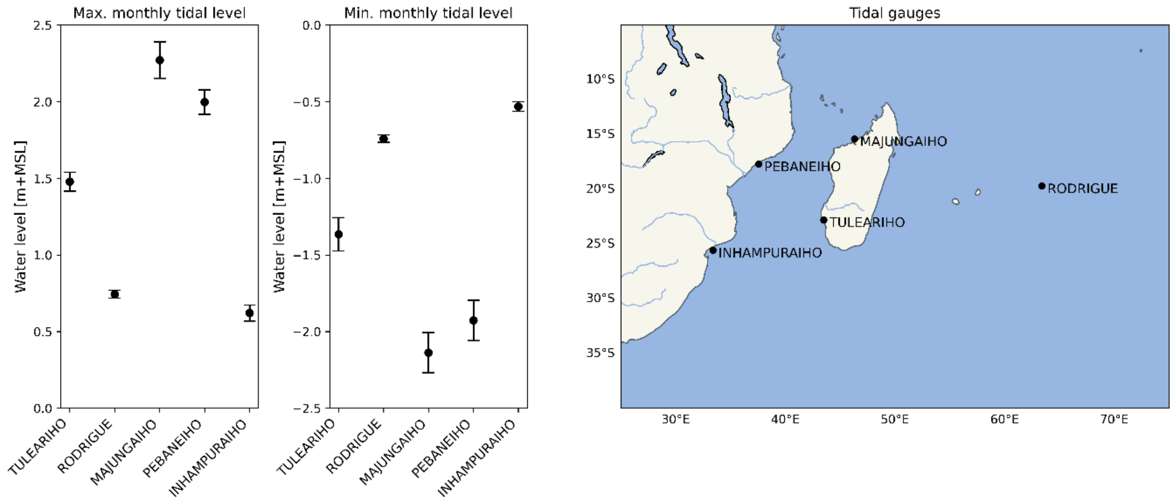

Supplementary Figure 1. Monthly variability of tidal levels. *Mean tidal level and standard deviation of the monthly maximum and minimum water levels, during the TC season in the east coast of Africa, for five IHO tide stations.*
